# Supplementary material for: Synthesis, Experimental and Theoretical Study of Azidochromones
Source: Molecules. 2022 Apr 20;27(9):2636. doi: 10.3390/molecules27092636 (PMC9105743; doi:10.3390/molecules27092636)
Supplement: Supplementary file 1 [file molecules-27-02636-s001.zip › molecules-1599380-SI.pdf]

molecules

Supplementary Materials

Synthesis, Experimental and Theoretical Study of Azidochromones

Ena G. Narváez-Ordoñez [1], Kevin A. Pabón-Carcelén [1], Daniel A. Zurita-Saltos [1], Pablo M. Bonilla-Valladares [1], Trosky G. Yánez-Darquea [1], Luis A. Ramos-Guerrero [2], Sonia E. Ulic [3,4], Jorge L. Jios [5], Gustavo A. Echeverría [6], Oscar E. Piro [6], Peter Langer [7,8], Christian D. Alcívar-León [1,*], and Jorge Heredia-Moya [9,*]

[1] Facultad de Ciencias Químicas, Universidad Central del Ecuador, Francisco Viteri s/n y Gilberto Gato Sobral, Quito 170521, Ecuador; egnarvaezo@uce.edu.ec (E.G.N.-O.); kevincarcelen@outlook.com (K.A.P.-C.); dazuritas@uce.edu.ec (D.A.Z.-S.); pmbonilla@uce.edu.ec (P.M.B.-V.); tgyanez@uce.edu.ec (T.G.Y.-D.).
[2] Centro de Investigación de Alimentos CIAL, Universidad UTE, Quito 170527, Ecuador; luis.ramos@ute.edu.ec.
[3] CEQUINOR (CONICET-UNLP), Facultad de Ciencias Exactas, Universidad Nacional de La Plata, Bv. 120 N° 1465 (1900) La Plata, Argentina; sonia@quimica.unlp.edu.ar.
[4] Departamento de Ciencias Básicas, Universidad Nacional de Luján, Facultad de Ciencias Exactas, Rutas 5 y 7, Luján 6700, Buenos Aires, Argentina.
[5] Laboratorio UPL (UNLP-CIC), Camino Centenario e/505 y 508 (1897) M.B. Gonnet and Departamento de Química, Facultad de Ciencias Exactas, Universidad Nacional de La Plata, República Argentina. 47 esq. 115, La Plata 1900, Argentina; jljios@quimica.unlp.edu.ar.
[6] Departamento de Física, Facultad de Ciencias Exactas, Universidad Nacional de La Plata e IFLP (CONICET, CCT-La Plata), C.C. 67, 1900, La Plata 1900, Argentina; geche@fisica.unlp.edu.ar (G.A.E.); piro@fisica.unlp.edu.ar (O.E.P.).
[7] Institut für Chemie, Universität Rostock, Albert-Einstein-Str. 3a, 18059, Rostock 18059, Germany; peter.langer@uni-rostock.de.
[8] Leibniz Institut für Katalyse, Universität Rostock e. V. (LIKAT), Albert-Einstein-Str. 29a, 18059, Rostock 18059, Germany.
[9] Centro de Investigación Biomédica (CENBIO), Facultad de Ciencias de la Salud Eugenio Espejo, Universidad UTE, Quito 170527, Ecuador.
* Correspondence: cdalcivar@uce.edu.ec (C.D.A.-L.); jorgeh.heredia@ute.edu.ec (J.H.-M.).

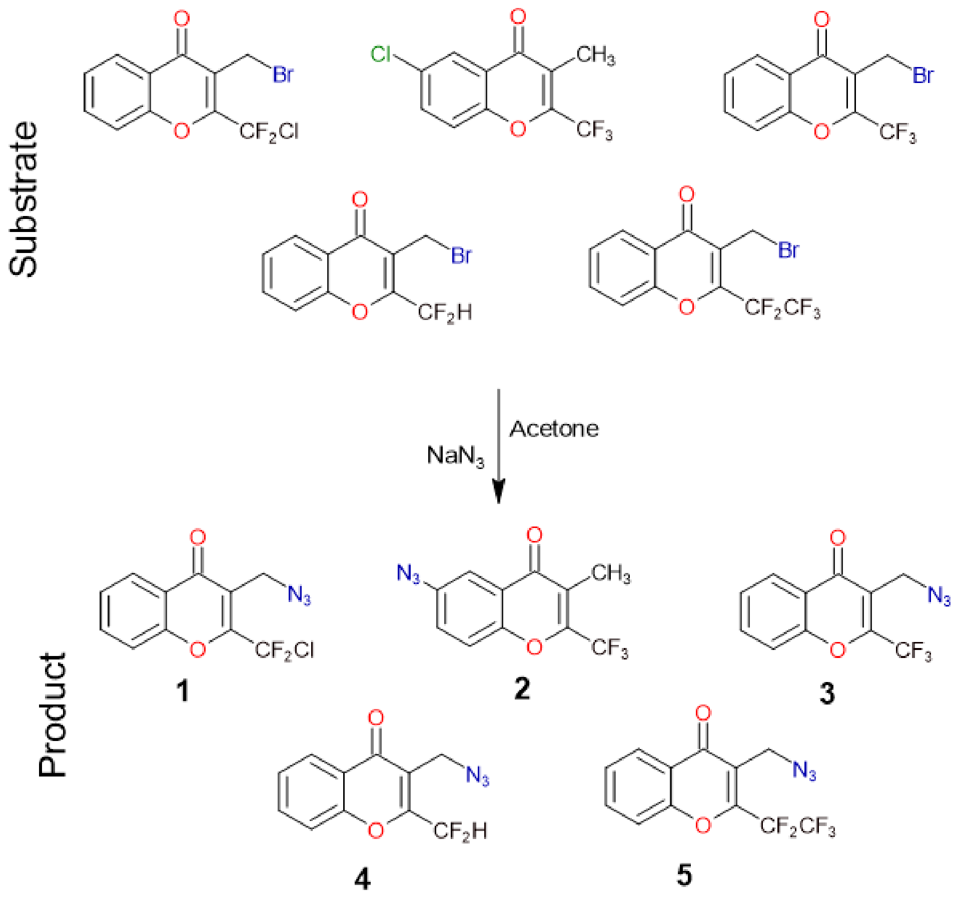

Scheme S1. General procedure of the synthesis of novel azidochromones.

Table S1. Reaction conditions for **1–5** compounds.

Title	Brominated substrate (mmol)	NaN_3 (mmol)	Acetone (mL)	Temperature (°C)	Time (h)	Yield (%)	Melting point (°C)
**1**	2.20	7.7	10	r.t	24	97	70.5–73.6
**2**	0.28	0.9	10	r.t	120	89	72–75.5
**3**	0.33	1.26	10	r.t	12	56	80–81
**4**	0.27	0.81	10	r.t	15	70	97.5–99.4
**5**	0.23	0.76	10	r.t	15	28	*

* : yellow oil

Conformational Study

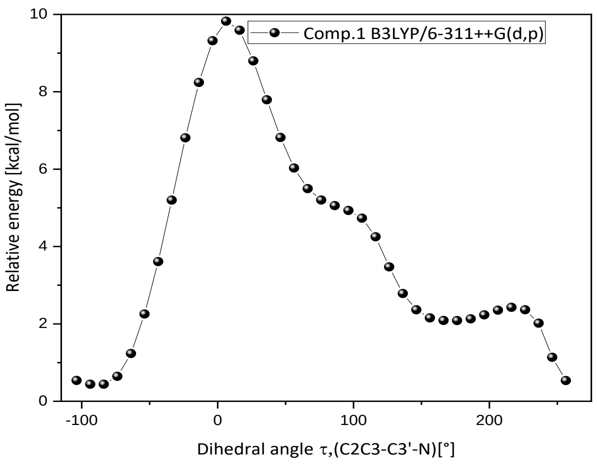

Figure S1. Potential energy curve for **1.**

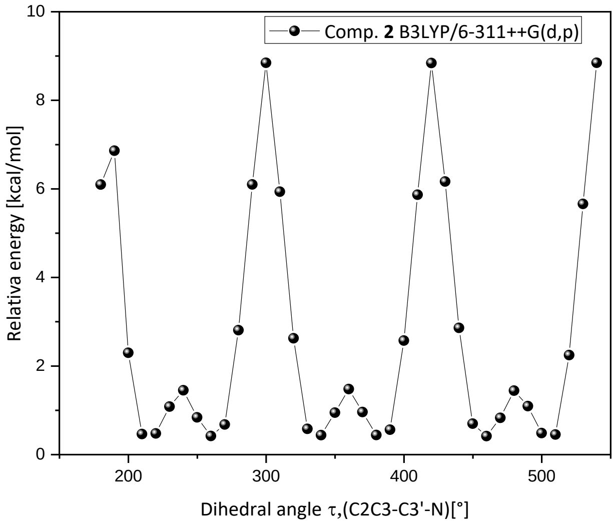

Figure S2. Potential energy curve for **2.**

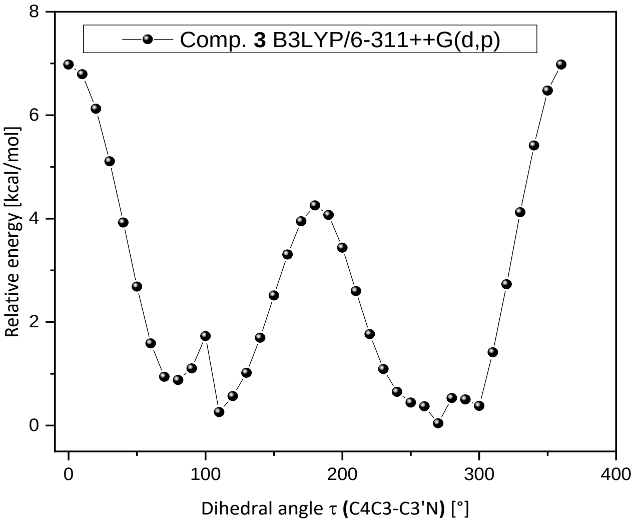

Figure S3. Potential energy curve for **3.**

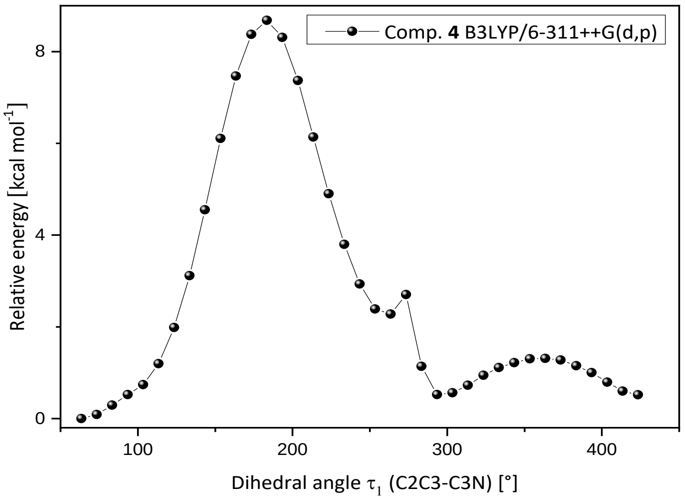

Figure S4. Potential energy curve for **4.**

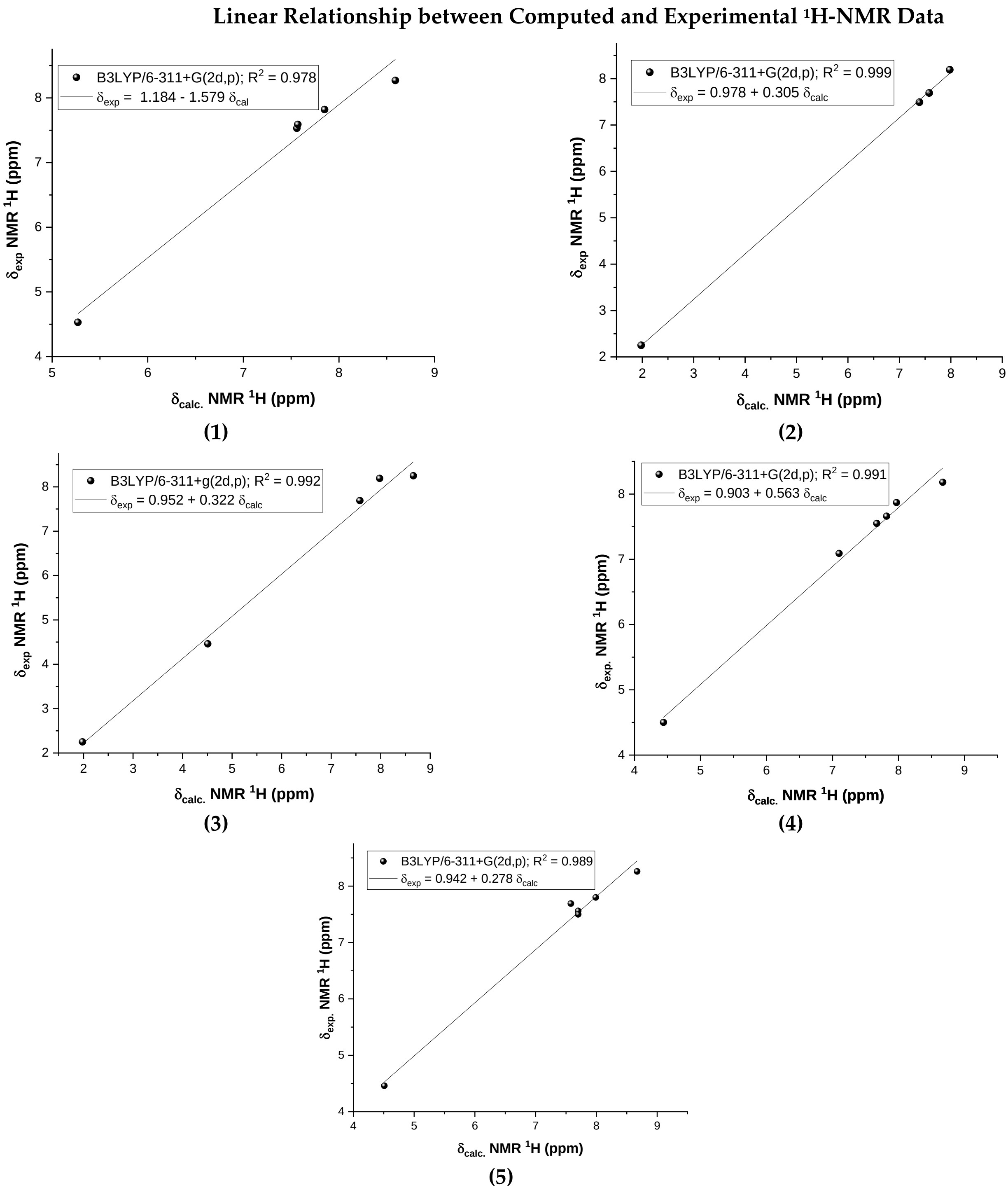

Figure S5. Linear relationship data ^{1}H-NMR **1–5.**

0 2 4 6 8 10

UV-Vis Spectra

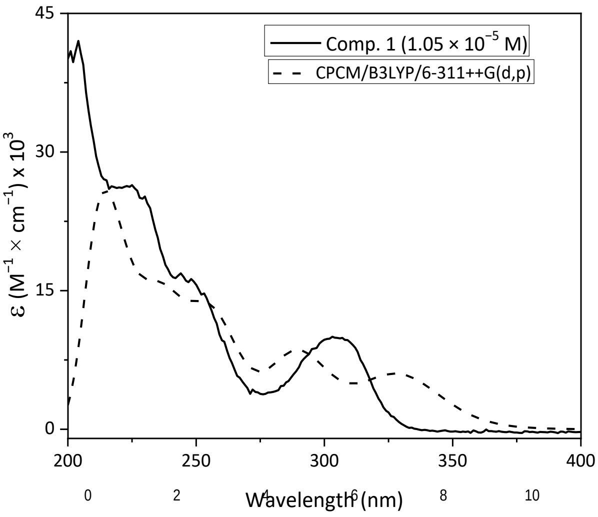

Figure S6. Experimental and calculated UV-Vis spectra of **1**.

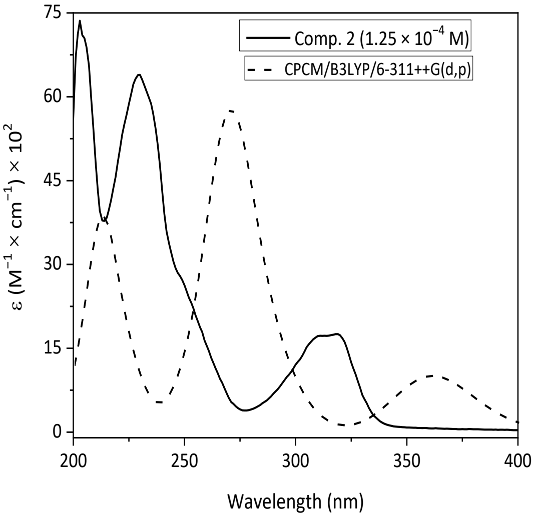

Figure S7. Experimental and calculated UV-Vis spectra of **2**.

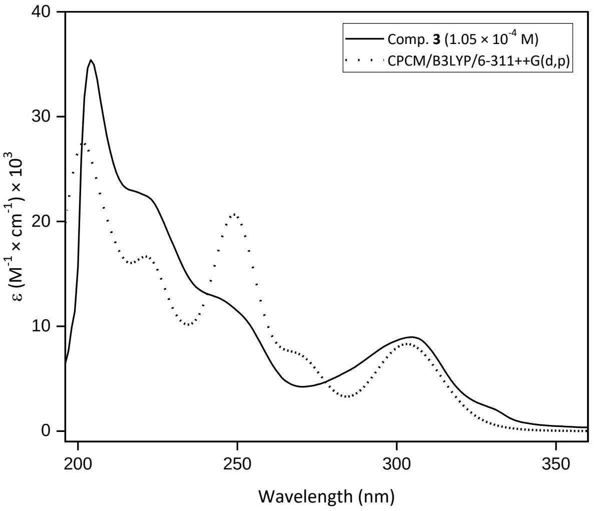

Figure S8. Experimental and calculated UV-Vis spectra of **3**.

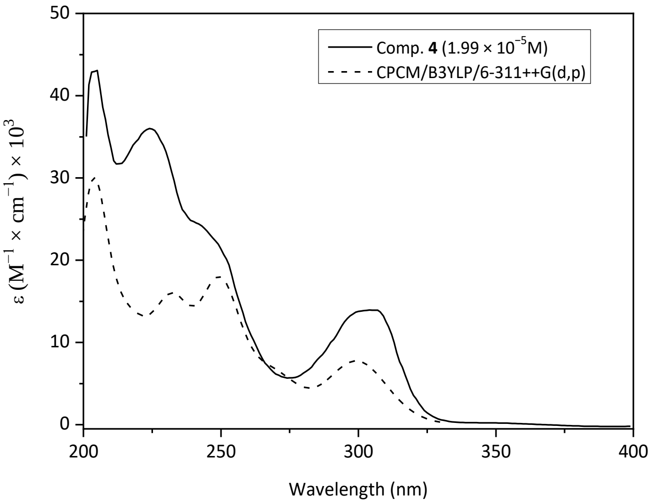

Figure S9. Experimental and calculated UV-Vis spectra of **4**.

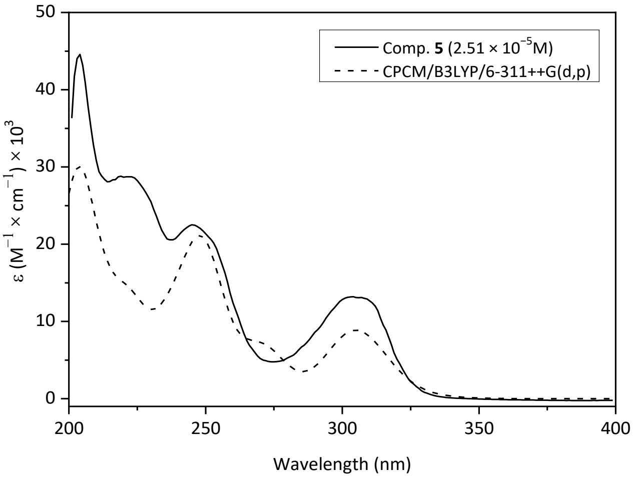

Figure S10. Experimental and calculated UV-Vis spectra of **5**.

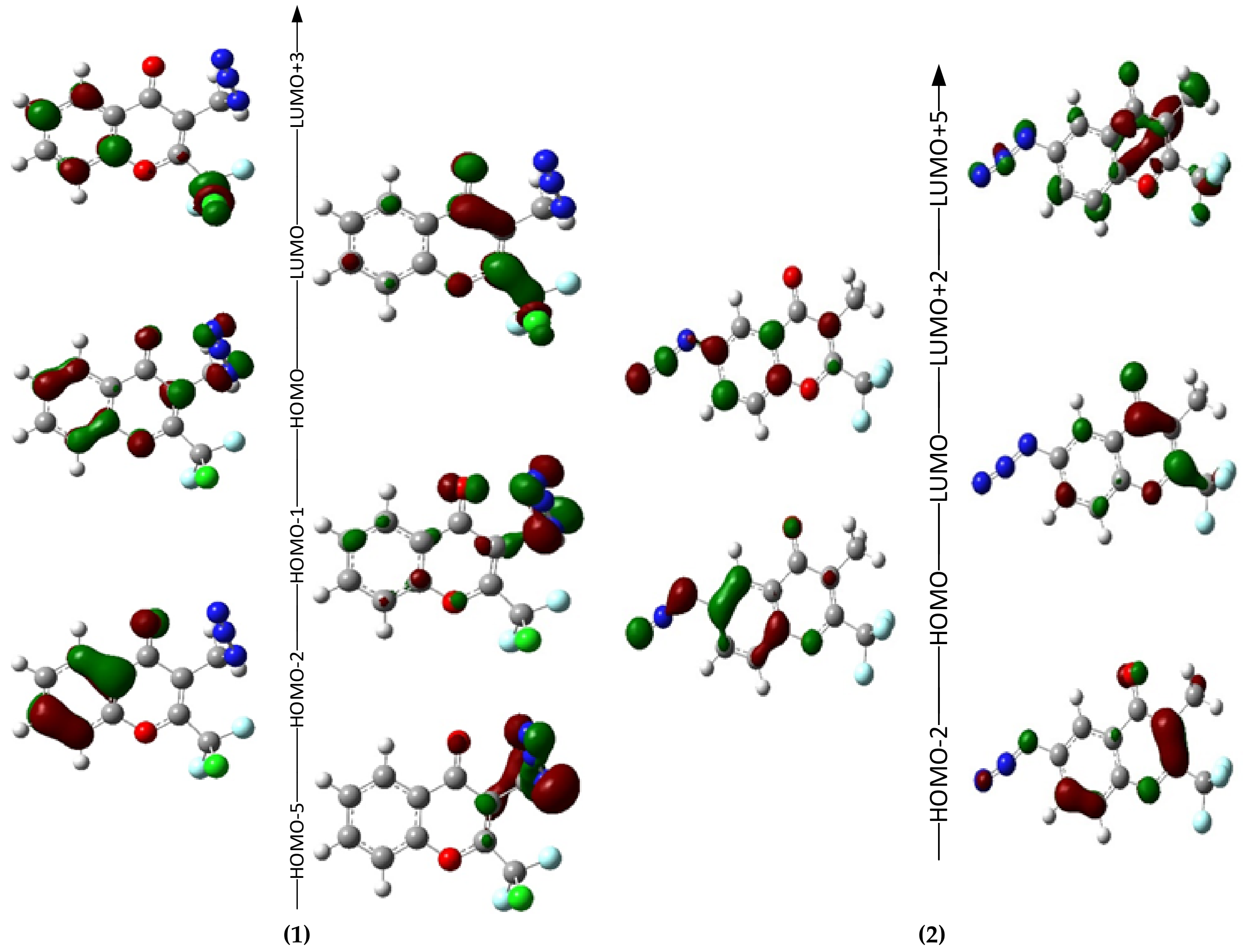

Figure S11. Molecular orbitals involved in the electronic transitions of **1** and **2**. The energy scale is only qualitative.

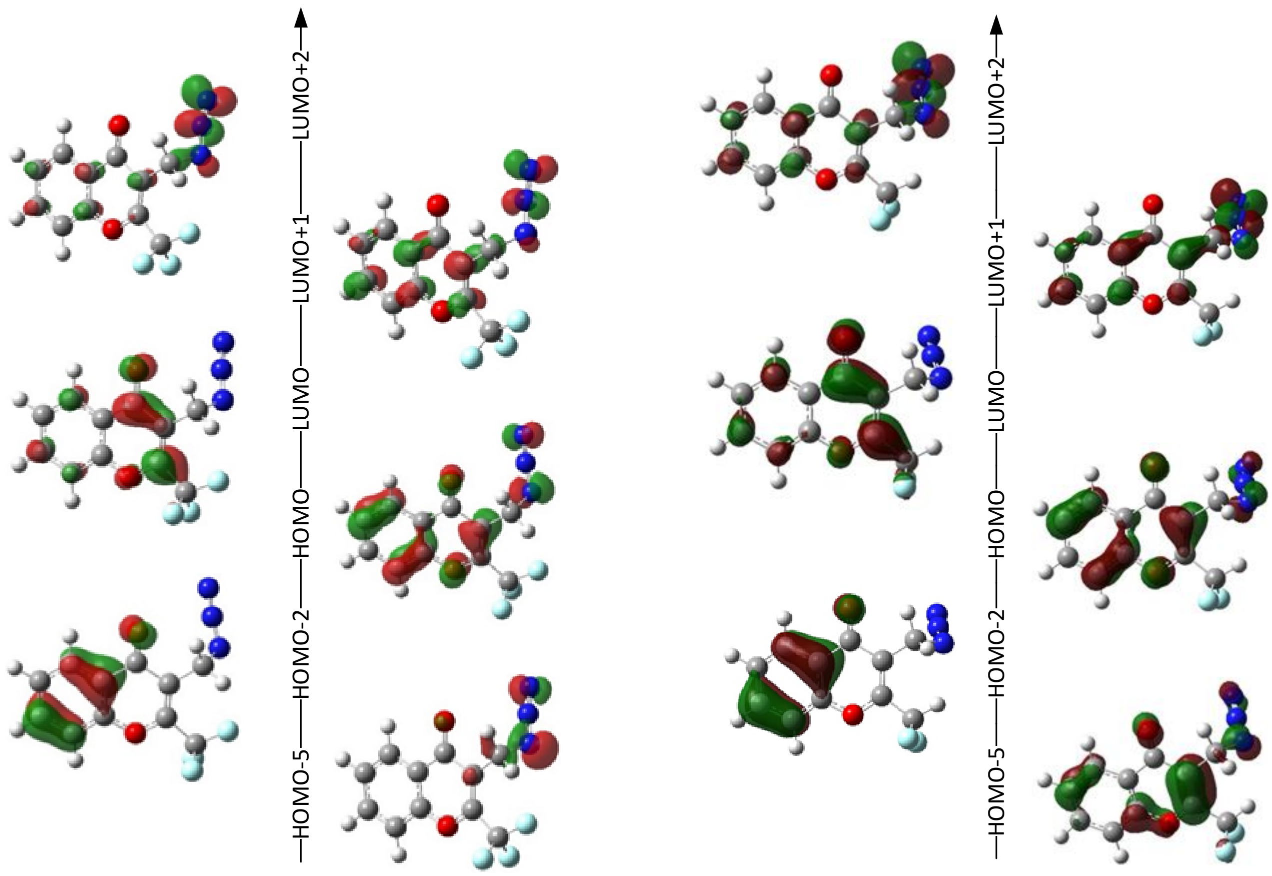

(3) **(4)**

Figure S12. Molecular orbitals involved in the electronic transitions of **3** and **4**. The energy scale is only qualitative.

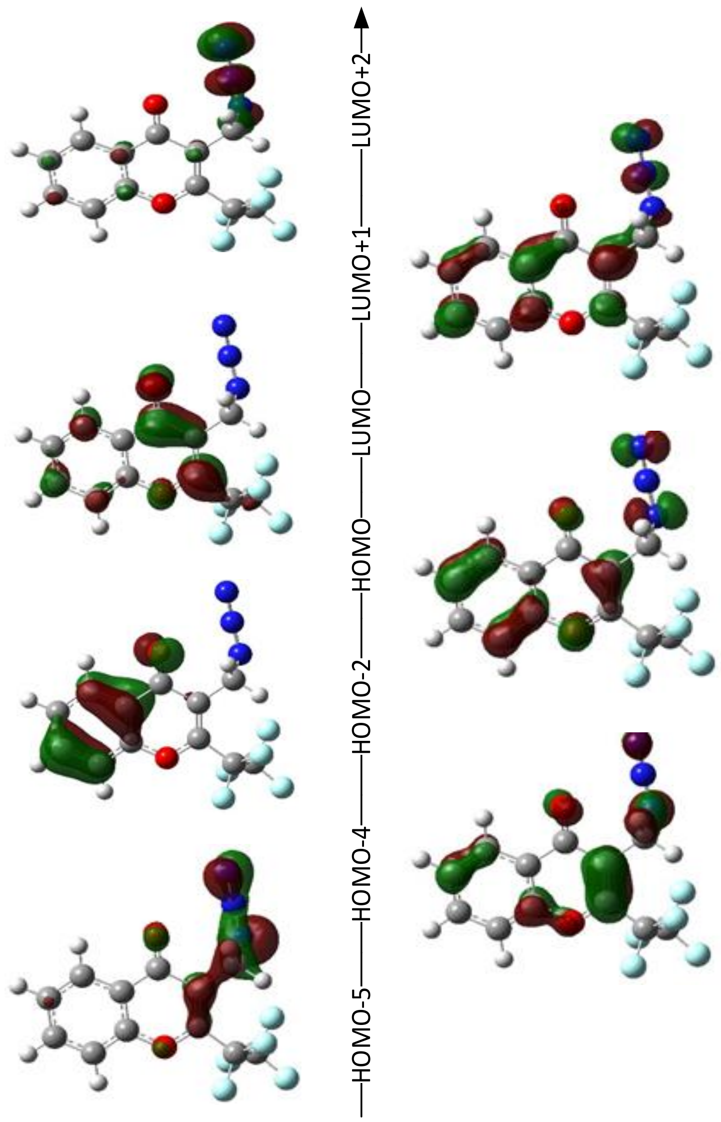

Figure S13. Molecular orbitals involved in the electronic transitions of **5**. The energy scale is only qualitative.

Crystallographic Data

Table S2. Crystal data and structure refinement results for 3-dibromomethyl-2-difluoromethyl chromone.

Parameters	Result
Empirical formula	$C_{11}H_7F_2N_3O_2$
Formula weight	251.20
Temperature	296(2) K
Wavelength	0.71073 Å
Crystal system	orthorhombic
Space group	Pbca
Unit cell dimensions	13.5520(5)
	7.1829(4)
	22.2110(9)
α/°	90
β/°	90

γ/°	90
Volume (Å3)	2162.1(2) Å3
Z	8
Density (calculated, Mg/cm3)	1.543
Absorption coefficient (mm-1)	0.132
F(000)/ mm3	1024.0
Crystal size	0.299 x 0.181 x 0.113 mm3
ϑ-range for data collection	3.338 to 26.466°.
Index ranges	−11 ≤ h ≤ 17, -6 ≤ k ≤ 8,−24 ≤ l ≤ 27
Reflections collected	6549
Independent reflections	2173 [R_{int} = 0.0260, R_{sigma} = 0.0274]
Observed reflections [I>2σ(I)]	1429
Completeness to ϑ = 71.98°	99.8 %
Refinement method	Complete least squares matrix in F^2
Data / restraints / parameters	2173/0/191
Goodness-of-fit on F^2	1.023
Final R indices [a] [I>2σ(I)]	R_1 = 0.0418, wR_2 = 0.0912
R indices (all data)	R_1 = 0.0732, wR_2 = 0.1078
Largest diff. peak and hole e Å-3	0.142 and -0.159 e.Å-3
Identification code	CCDC 2119625

[a] $R_1=\Sigma||F_o|-|F_c||/\Sigma|F_o|$, $wR_2=[\Sigma w(|F_o|^2-|F_c|^2)^2/\Sigma w(|F_o|^2)^2]^{1/2.}$

Crystallographic Structural Results

Table S3. Bond lengths [Å] and angles [°] for 3-azidomethyl-2-difluoromethyl chromone **(4).**

Type bond	Exp.	Calc.
r (O1–C2)	1.355 (2)	1.3462
r (C2–C3)	1.339 (3)	1.3530
r (C3–C4)	1.462 (3)	1.4776
r (C4–C4a)	1.468 (3)	1.4743
r (C4a–C5)	1.398 (3)	1.4048
r (C5–C6)	1.363 (3)	1.3830
r (C6–C7)	1.388 (4)	1.4042
r (C7–C8)	1.363 (4)	1.3852
r (C8–C8a)	1.389 (3)	1.3956
r (C8a–O1)	1.375 (2)	1.3702
r (C3–C3′)	1506 (3)	1.5151
r (C3′–N1)	1.478 (3)	1.4866
r (N1–N2)	1.221 (2)	1.2339
r (N2–N3)	1.126 (3)	1.1327
r (C4–O2)	1.227 (2)	1.2252
r (C2–C2′)	1.495 (3)	1.5105
r (C2′–F1)	1.363 (3)	1.3654
r (C2′–F2)	1.351 (2)	1.3724

Table S4. Angles [°] for 3-azidomethyl-2-difluoromethyl chromone **(4).**

Atoms	Angle exp. [°]	Angle calc. [°]	Atoms	Angle calc. [°]
C5–C6–C7	120.2 (2)	120.03	C2–C3–C4	119.40
C6–C5–C4a	120.4 (3)	120.36	C2–C3–C3′	123.13
C2–C3–C3	117.8 (2)	123.13	C4–C3–C3′	117.47
C5–C4a–C8a	119.91 (17)	118.43	C3–C2–O1	124.73
C4–C4a–C8a	122.3 (2)	120.01	C3–C2–C2′	124.54
O1–C8a–C4a	121.80 (17)	121.39	C2′–C2–O1	110.73
O1–C8a–C8	115.66 (19)	116.71	F2–C2′–F1	107.23
C4a–C8a–C8	122.5 (2)	121.90	F2–C2′–C2	109.51
C7–C8–C8a	117.8 (2)	118.52	F1–C2′–C2	109.99
C8–C7–C6	121.3 (2)	120.75	N1–C3′–C3	114.00
O2–C4–C3	121.84 (18)	122.25	N2–N1–C3′	116.04
O2–C4–C4a	123.17 (18)	122.95	N3–N2–N1	172.86
C3–C4–C4a	114.99 (17)	114.79	C2–O1–C8a	119.66

Table S5. Fractional Atomic Coordinates (×10^4) and Equivalent Isotropic displacement Parameters (Å^2×10^3) for **4**. U_{eq} is defined as 1/3 of the trace of the orthogonalised U_{IJ} tensor.

Atom	x	y	z	U(eq)
C6	9083(2)	3749(4)	5535.6(14)	75.7(8)
C5	8785.8(17)	4132(3)	6108.6(12)	60.5(6)
C4a	7781.5(14)	4232(2)	6248.1(9)	45.5(5)
C8a	7113.1(14)	3915(3)	5789.9(9)	46.8(5)
C8	7404(2)	3524(3)	5203.8(10)	62.8(6)
C7	8390(2)	3446(4)	5086.1(13)	77.1(8)
C4	7426.7(15)	4642(3)	6858.5(9)	46.6(5)
C3	6354.4(14)	4775(3)	6918.3(8)	43.6(5)
C2	5776.1(14)	4407(3)	6444.0(9)	45.0(5)
C2′	4673.1(16)	4345(3)	6440.9(11)	56.5(6)
C3′	5958.1(18)	5330(3)	7525.7(10)	53.8(5)
N1	5937.8(12)	3727(3)	7943.0(7)	55.2(5)
N2	6602.4(14)	3717(3)	8314.5(9)	64.5(5)
N3	7172.9(19)	3561(4)	8679.4(12)	118.6(11)
O1	6111.4(10)	3969.2(19)	5887.0(6)	50.7(4)
O2	7979.7(10)	4868(2)	7289.6(6)	66.8(4)
F1	4389.1(9)	2573(2)	6306.4(6)	73.0(4)
F2	4313.1(9)	5430(2)	5995.3(6)	79.8(4)

Table S6. Selected intermolecular contacts [Å and °] for **4.**

D–X···A	d(D–X)	d(X···A)	d(D···A)	∠ (D–H···A)	R **	E_{ele}	E_{pol}	E_{dis}	E_{rep}	E_{tot}
**C7–H7···F2** i	0.920	2.540	3.455	173	10.08	−3.6	−0.7	−7.5	5.7	−7.3
**C3′–H3′A···F2** ii	0.963	2.669	3.090	107	6.96	−7.1	−1.7	−23.4	12.9	−21.2
**C8–H8···F1** iii	0.978	2.731	3.616	151	8.43	−2.7	−0.4	−7.6	3.1	−7.9
**C2′–H···O2** iv	0.971	2.724	3.655	161	7.88	−11.4	−2.8	−16.6	11.2	−21.6
**C5–H5···N1** v	0.897	2.779	3.609	154	7.88	−11.4	−2.8	−16.6	11.2	−21.6
**C2′–F1···N2** vi	1.351	3.076	3.626	103	6.96	−7.1	−1.7	−23.4	12.9	−21.2
**C2′–F2···N3** vii	1.363	3.083	3.445	93	7.88	−11.4	−2.8	−16.6	11.2	−21.6

Symmetry transformations used to generate equivalent atoms: (i) 1/2+x,1/2−y,1−z; (ii) 1−x,−1/2+y,1.5−z; (iii) −x,−y,1−z; (iv) −1/2+x,y,1/2−z; (v) 1/2+x,y,1/2−z; (vi) −x,1/2+y,1/2−z; (vii) 1/2+x,y,1/2−z. ** Distance between molecular centroids (mean atomic position) in Å.

Table S7. Geometrical parameters for the π-stacking moieties involved in the π···π interactions of **4** (Å, º).

Rings I – J [a]	*Cg(I)· · Cg(J)* [b]	*Cg(I)· · ·Perp* [c]	*Cg(J)· · ·Perp* [d]	*α* [e]	*β* [f]	*γ* [g]	symmetry
**Cg(2)·· ·Cg(3)**	3.5691 (2)	3.4996	3.4974	1	12.4	13.6	1/2−x,−1/2+y,z
**Cg(1)·· ·Cg(2)**	3.5943 (2)	3.4941	3.5107	1	16.9	17.8	1/2−x,−1/2+y,z
**Cg(3)·· ·Cg(3)**	3.5976 (2)	3.5022	3.5038	0	11.3	11.5	1/2−x,1/2+y,z
**Cg(1)·· ·Cg(3)**	3.6739 (2)	3.4990	3.5157	0	13.1	13.2	1/2−x,−1/2+y,z

[a] Cg(1), Cg(2), Cg(3) are the centroids of pyrane, benzene and chromone rings, respectively. [b] Centroid distance between ring i and ring J. [c] Perpendicular distance of Cg(I) on ring J (Å). [d] Perpendicular distance of Cg(J) on ring I (Å). [e] Dihedral Angle between Planes I and J (Degrees). [f] Angle between the centroid vector Cg(i)· · ·Cg(j) and the normal to the plane (i). [g] Angle between the centroid vector Cg(i)· · ·Cg(j) and the normal to the plane (j).

Table S8. Geometrical parameters of C-O···π interactions* for **4** (Å, º).

Item	X···Cg[a]	X-perp [b]	γ [c]	∠ C-X···Cg(j)	Symmetry
C4-O2···Cg1	3.8276(2)	3.588	20.4	75	1/2−x,−1/2+y,z

* (H···Cg < Å, O···Cg y F···Cg < Å, γ < o). [a] Centroid of rings. [b] Perpendicular distance of X to ring plane J. [c] Angle between the Cg-X vector and ring J normal.

Table S9. Interaction energies (kJ/mol) for **4.**

Interaction Molecule Color	N	Symop	R	Electron Density	E_elec.	E_pol.	E_dis.	E_rep.	E_tot.
	2	−x+1/2, y+1/2, z	4.31	B3LYP/6-31G(d,p)	−6.9	−2.4	−57.4	26.4	−42.7
	2	−x, y+1/2, −z+1/2	6.96	B3LYP/6-31G(d,p)	−7.1	−1.7	−23.4	12.9	−21.2
	2	x+1/2, −y+1/2, −z	10.08	B3LYP/6-31G(d,p)	−3.6	−0.7	−7.5	5.7	−7.3
	2	x+1/2, y, −z+1/2	7.88	B3LYP/6-31G(d,p)	−11.4	−2.8	−16.6	11.2	−21.6
	1	−x, −y, −z	8.43	B3LYP/6-31G(d,p)	−2.7	−0.4	−7.6	3.1	−7.9
	2	−x+1/2, −y, z+1/2	11.43	B3LYP/6-31G(d,p)	−0.6	−0.4	−3.2	0.6	−3.4
	2	x, −y+1/2, z+1/2	11.35	B3LYP/6-31G(d,p)	−4.2	−0.6	−3.6	1.6	−7.1
	1	−x, −y, −z	11.64	B3LYP/6-31G(d,p)	0.5	−0.5	−6.7	2.9	−3.9

Table S10. Scale factors for benchmarked energy models. See Mackenzie et al. IUCrJ (2017).

Energy Model	k_ele	k_pol	k_disp	k_rep
CE-HF ... HF/3-21G electron densities	1.019	0.651	0.901	0.811
CE-B3LYP ... B3LYP/6-31G(d,p) electron densities	1.057	0.740	0.871	0.618

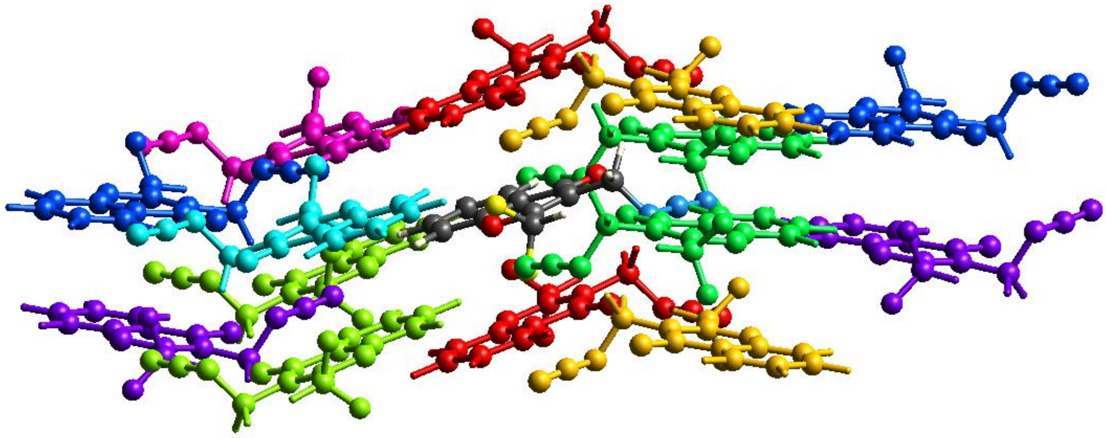

Figure S14. Interaction energies for **4**.

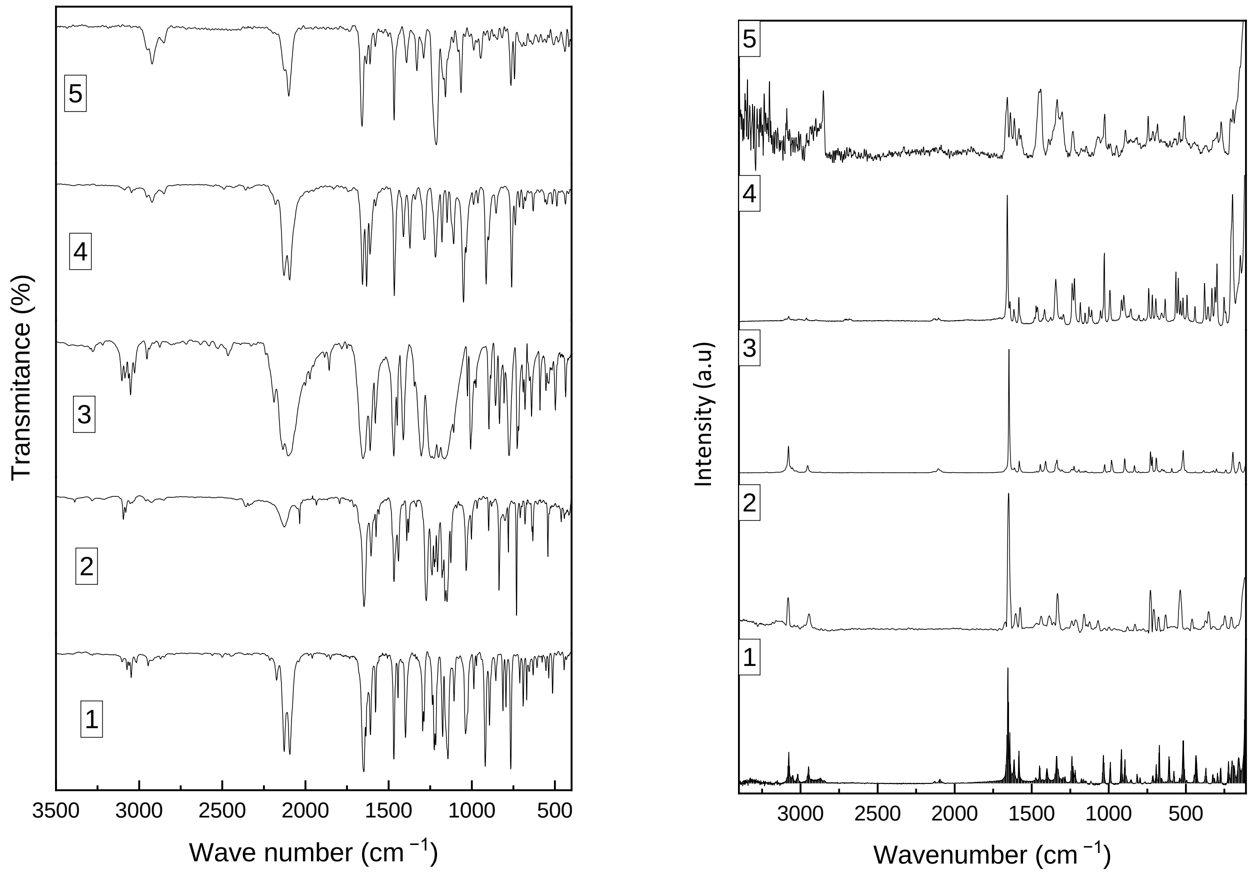

(**a**) (**b**)

Figure S15. (**a**) IR and (**b**) Raman spectra of **1**–**5**.

NMR Spectra

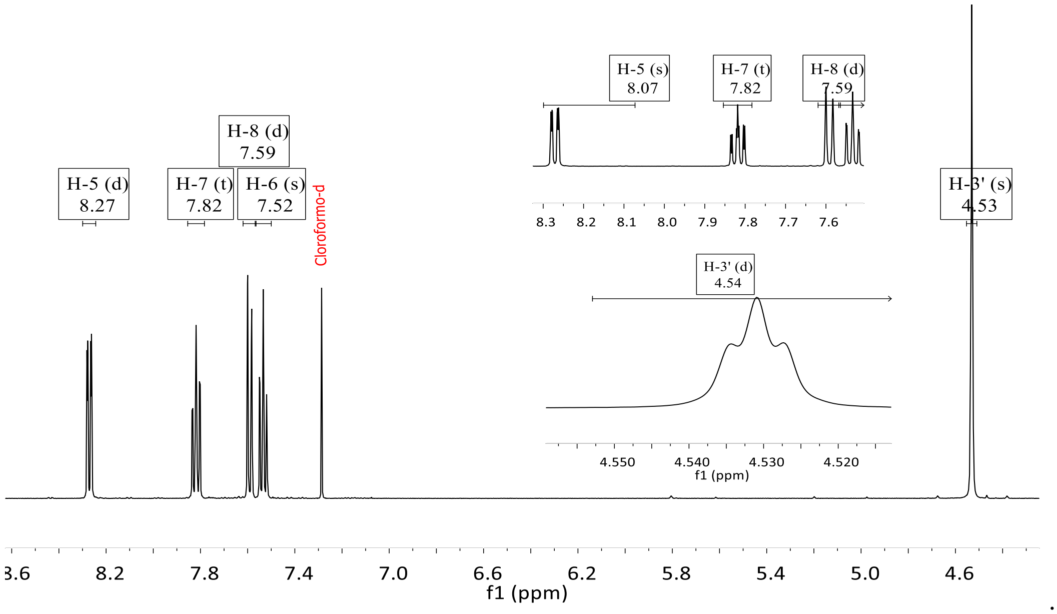

(a)

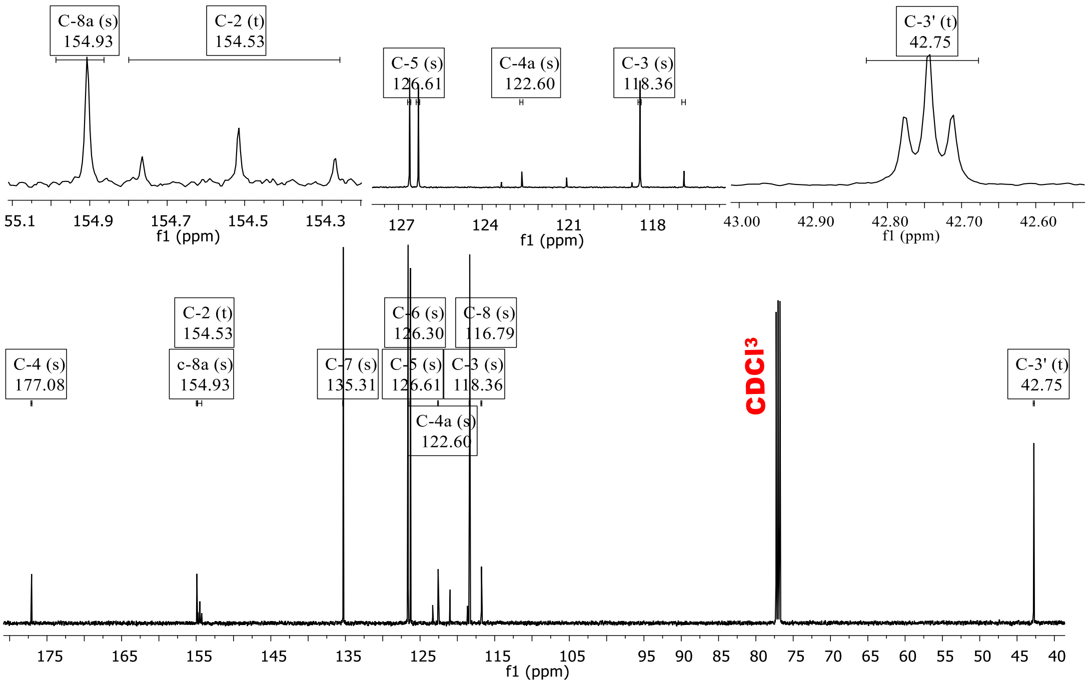

(b)

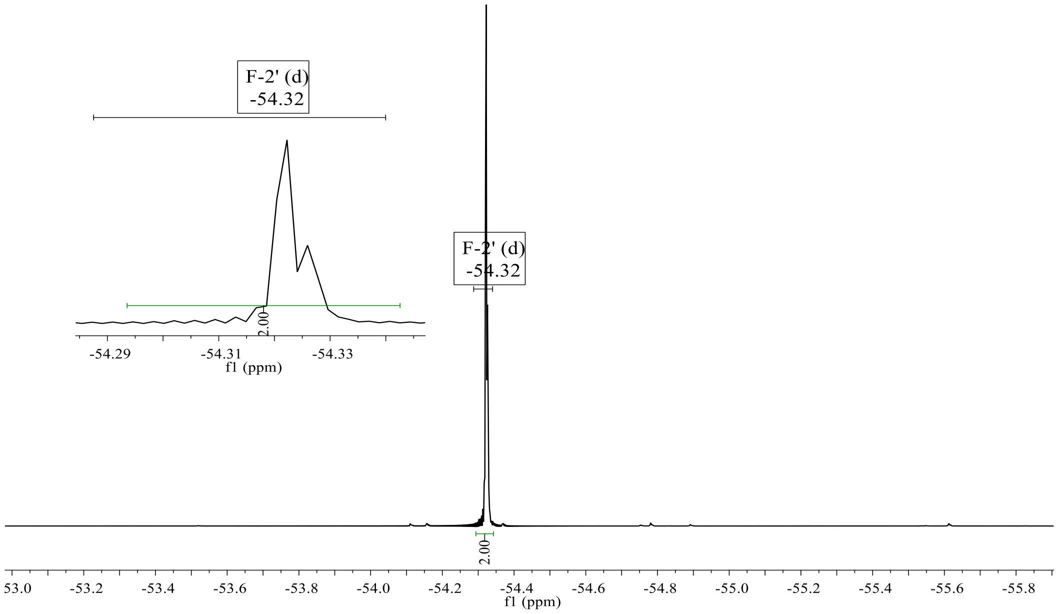

(c)

Figure S16. Compound **1** (**a**) ^{1}H-NMR, (**b**) ^{13}C-NMR and (**c**) ^{19}F-NMR.

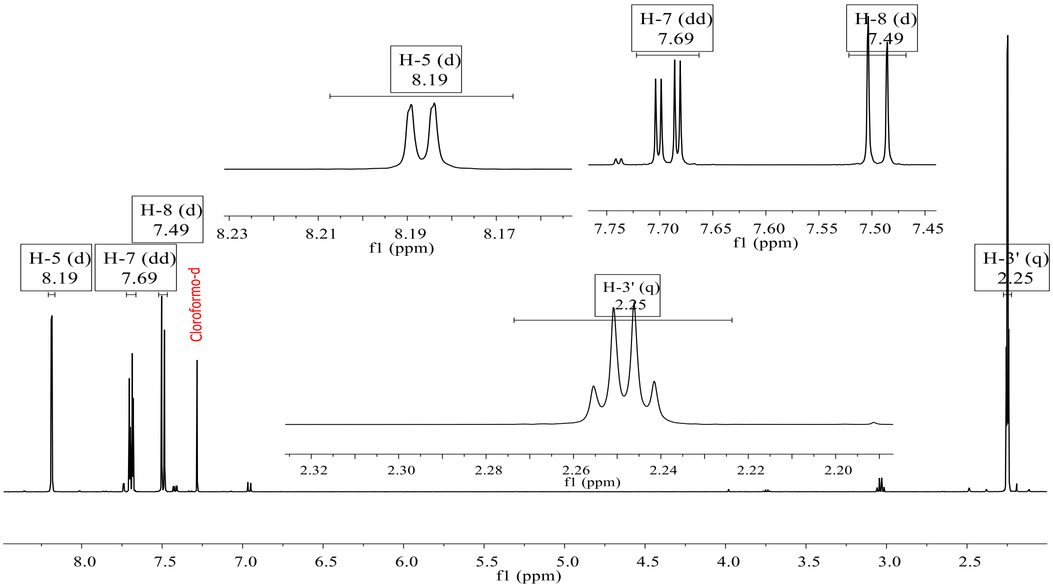

(a)

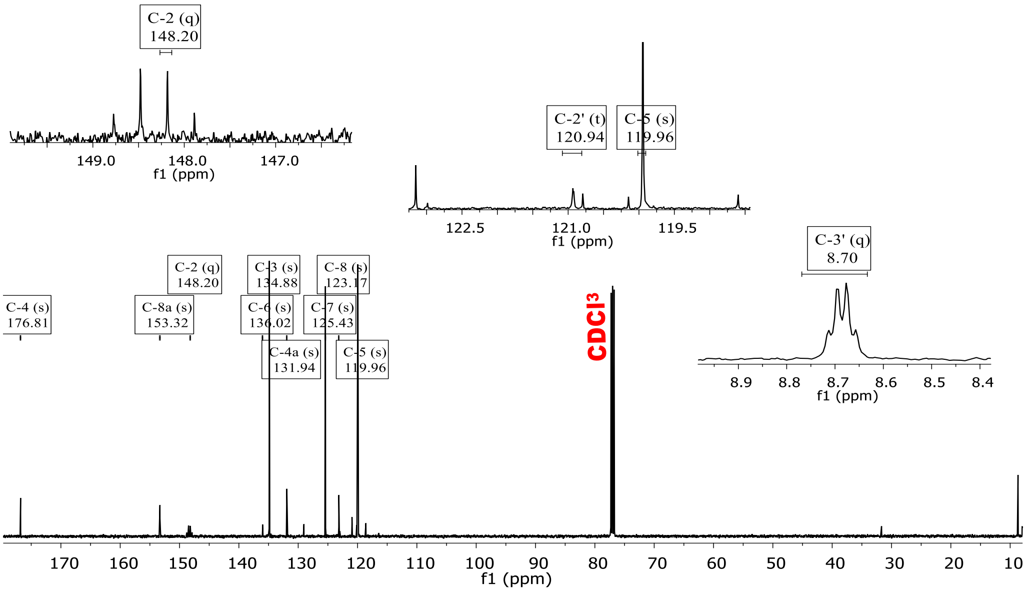

(b)

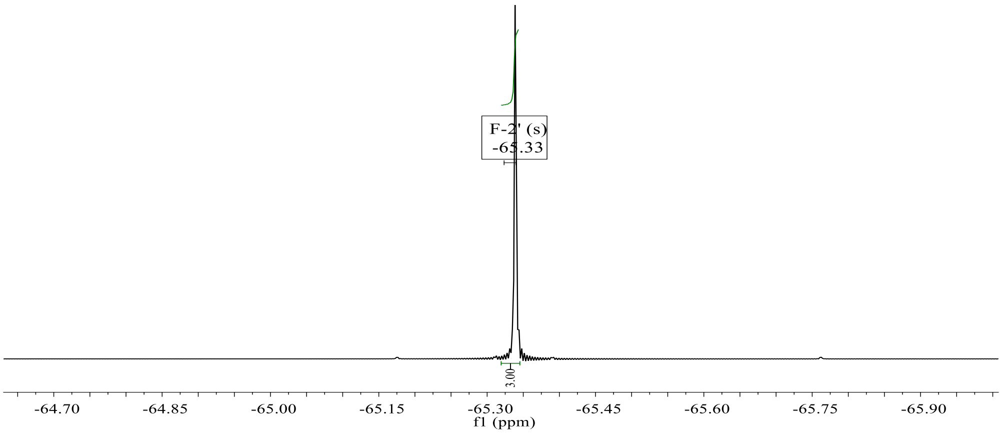

(c)

Figure S17. Compound **2** **(a)** ^{1}H-NMR, **(b)** ^{13}C-NMR and **(c)** ^{19}F-NMR.

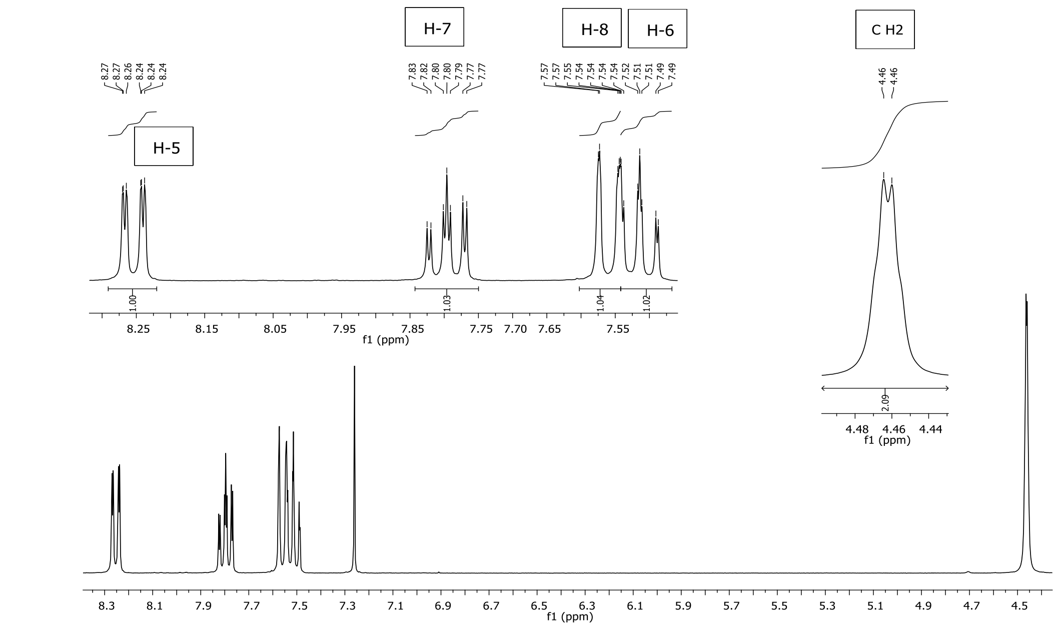

(a)

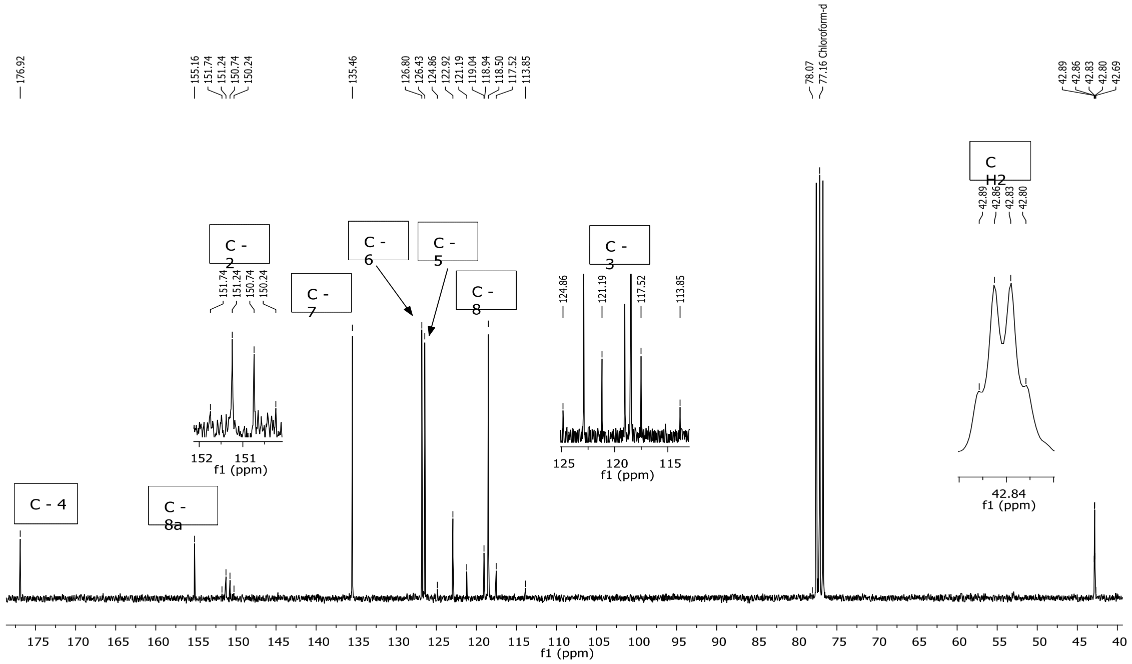

(b)

Figure S18. Compound **3** **(a)** ^{1}H-NMR, **(b)** ^{13}C-NMR and **(c)** ^{19}F-NMR.

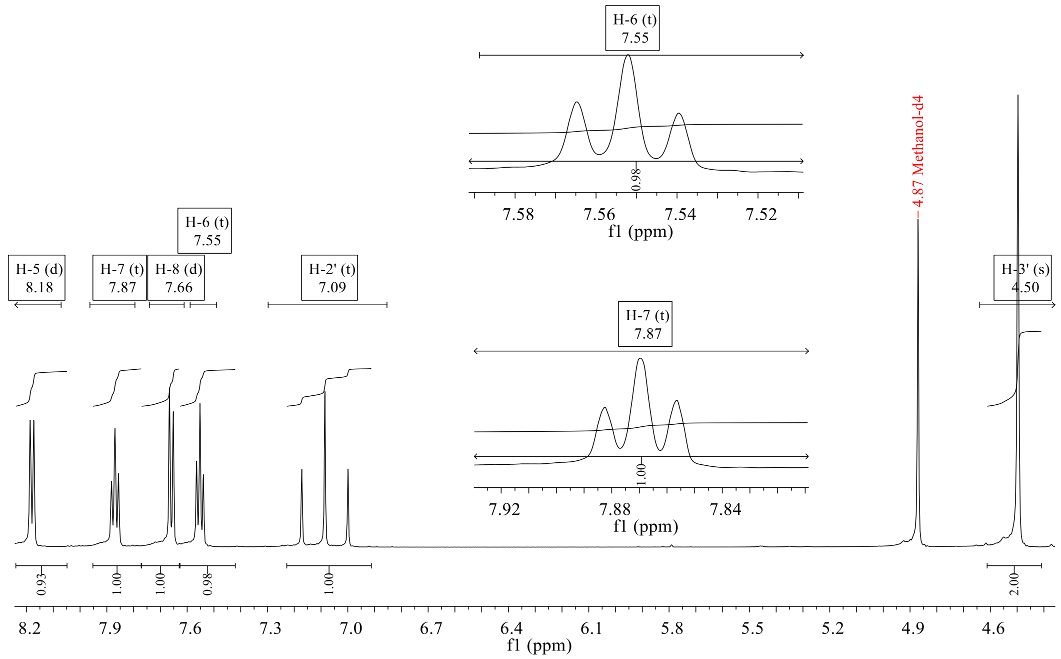

(a)

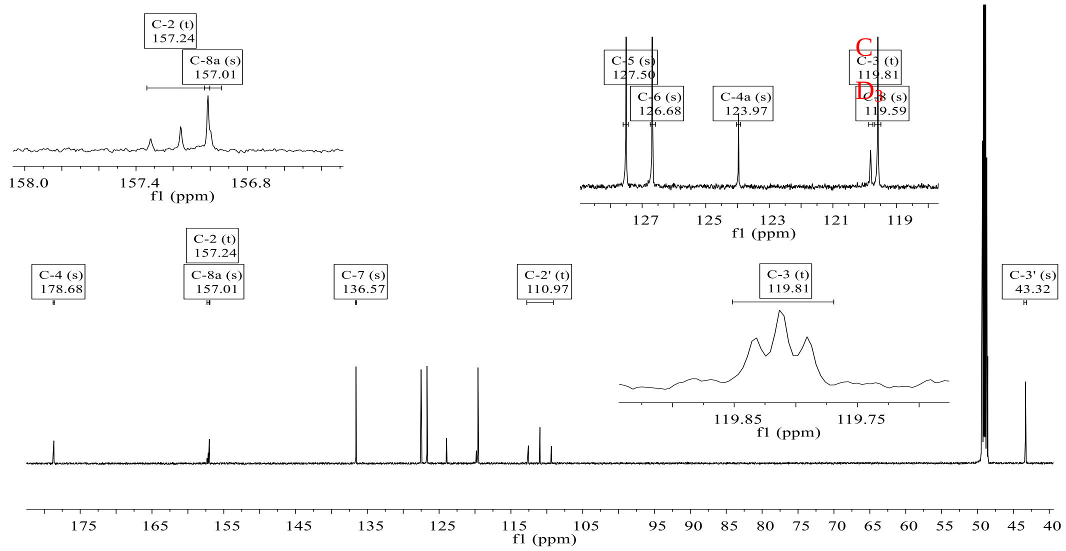

(b)

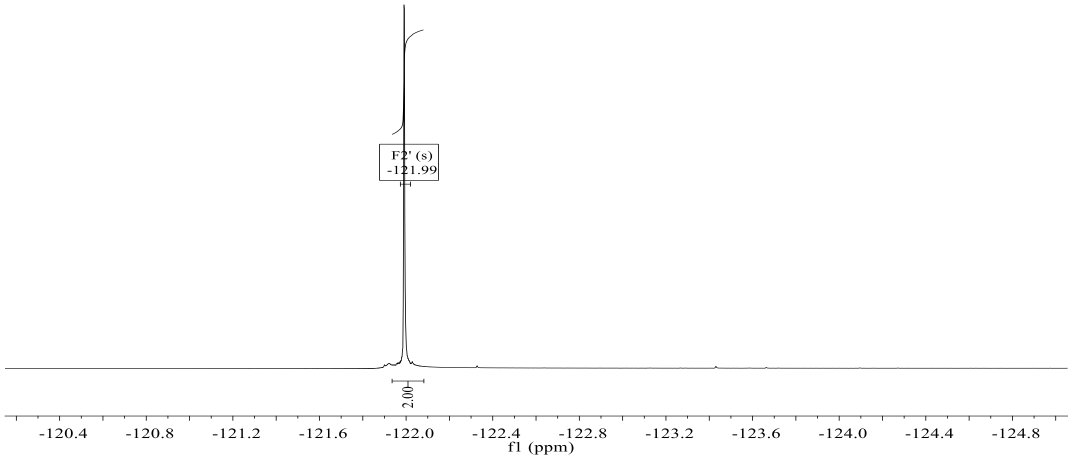

(c)

Figure S19. Compound **4** **(a)** ^{1}H-NMR, **(b)** ^{13}C-NMR and **(c)** ^{19}F-NMR.

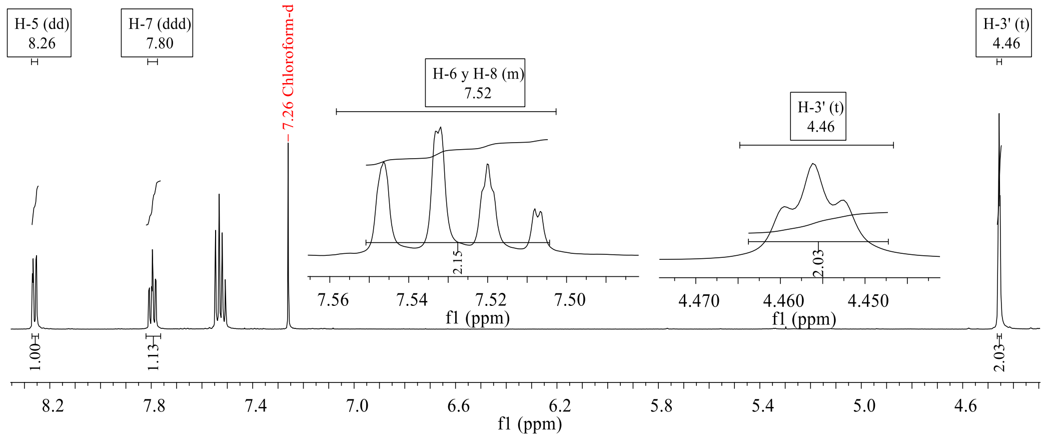

(a)

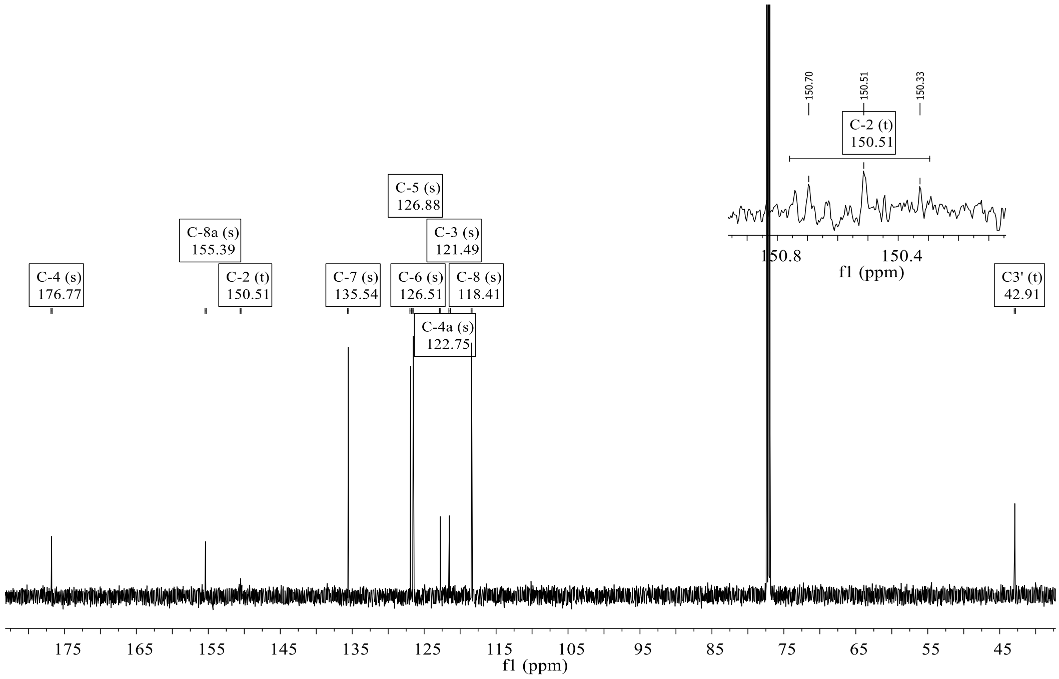

(b)

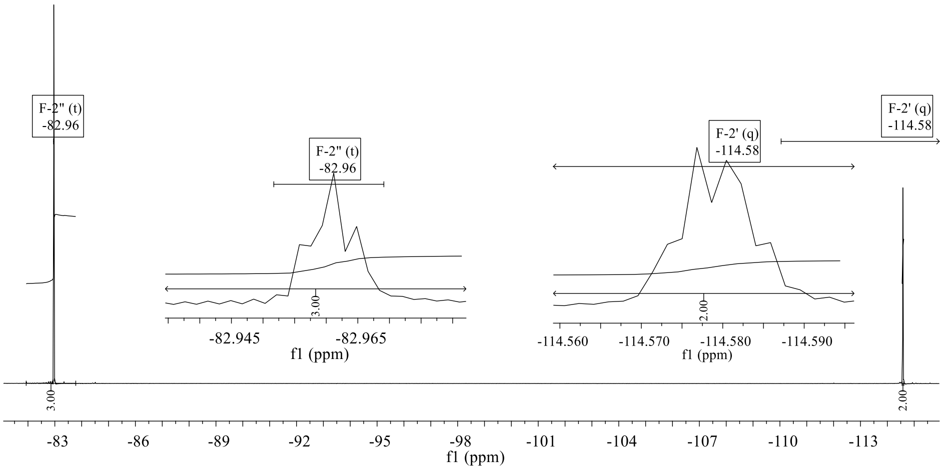

(c)

Figure S20. Compound **5** **(a)** ^{1}H-NMR, **(b)** ^{13}C-NMR and **(c)** ^{19}F-NMR.

Mass Spectra

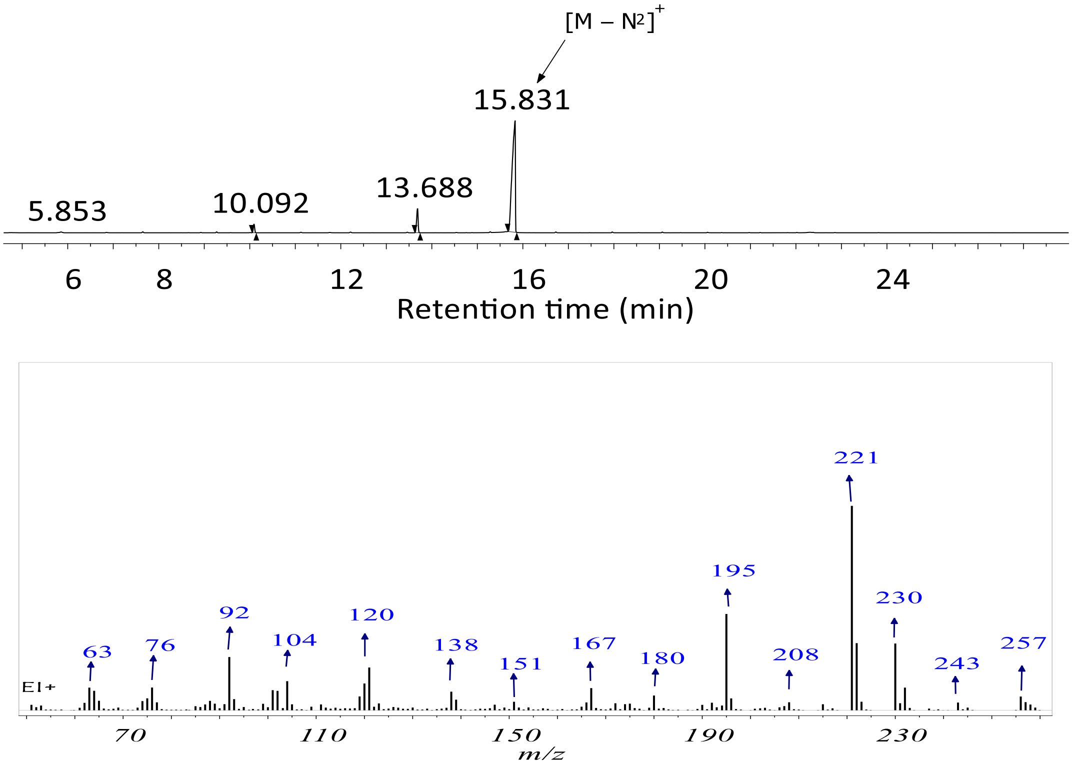

Figure S21. GS-MS of **1**.

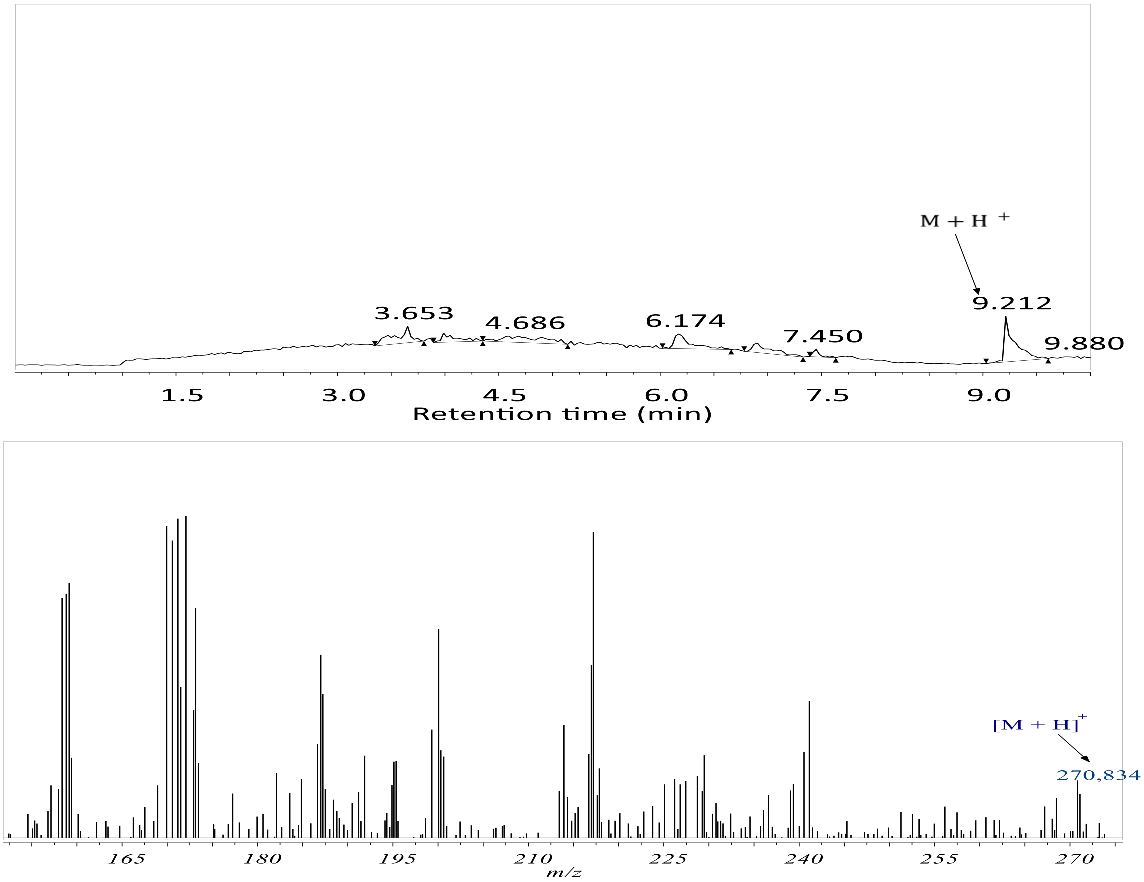

Figure S22. HPLC-EM of **2**.

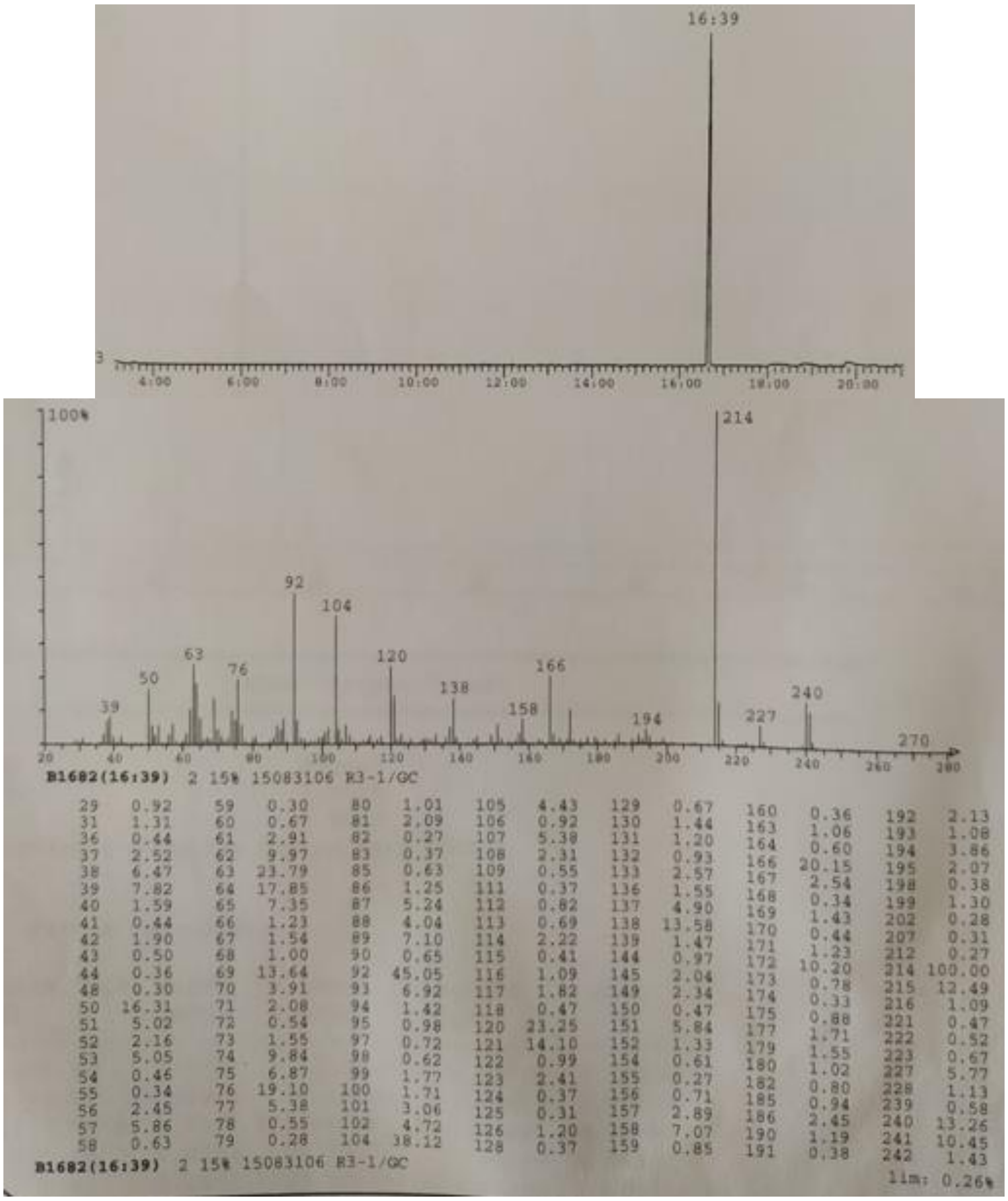

29	0.92	59	0.30	80	1.01	105	4.43	129	0.67	160	0.36	192	2.13
31	1.31	60	0.67	81	2.09	106	0.92	130	1.44	163	1.06	193	1.08
36	0.44	61	2.91	82	0.27	107	5.38	131	1.20	164	0.60	194	3.86
37	2.52	62	9.97	83	0.37	108	2.31	132	0.93	166	20.15	195	2.07
38	6.47	63	23.79	85	0.63	109	0.55	133	2.57	167	2.54	198	0.38
39	7.82	64	17.85	86	1.25	111	0.37	136	1.55	168	0.34	199	1.30
40	1.59	65	7.35	87	5.24	112	0.82	137	4.90	169	1.43	202	0.28
41	0.44	66	1.23	88	4.04	113	0.69	138	13.58	170	0.44	207	0.31
42	1.90	67	1.54	89	7.10	114	2.22	139	1.47	171	1.23	212	0.27
43	0.50	68	1.00	90	0.65	115	0.41	144	0.97	172	10.20	214	100.00
44	0.36	69	13.64	92	45.05	116	1.09	145	2.04	173	0.78	215	12.49
48	0.30	70	3.91	93	6.92	117	1.82	149	2.34	174	0.33	216	1.09
50	16.31	71	2.08	94	1.42	118	0.47	150	0.47	175	0.88	221	0.47
51	5.02	72	0.54	95	0.98	120	23.25	151	5.84	177	1.71	222	0.52
52	2.16	73	1.55	97	0.72	121	14.10	152	1.33	179	1.55	223	0.67
53	5.05	74	9.84	98	0.62	122	0.99	154	0.61	180	1.02	227	5.77
54	0.46	75	6.87	99	1.77	123	2.41	155	0.27	182	0.80	228	1.13
55	0.34	76	19.10	100	1.71	124	0.37	156	0.71	185	0.94	239	0.58
56	2.45	77	5.38	101	3.06	125	0.31	157	2.89	186	2.45	240	13.26
57	5.86	78	0.55	102	4.72	126	1.20	158	7.07	190	1.19	241	10.45
58	0.63	79	0.28	104	38.12	128	0.37	159	0.85	191	0.38	242	1.43

B1682(16:39) 2 15% 15083106 R3-1/GC

lim: 0.26%

Figure S23. GC-MS of **3**.

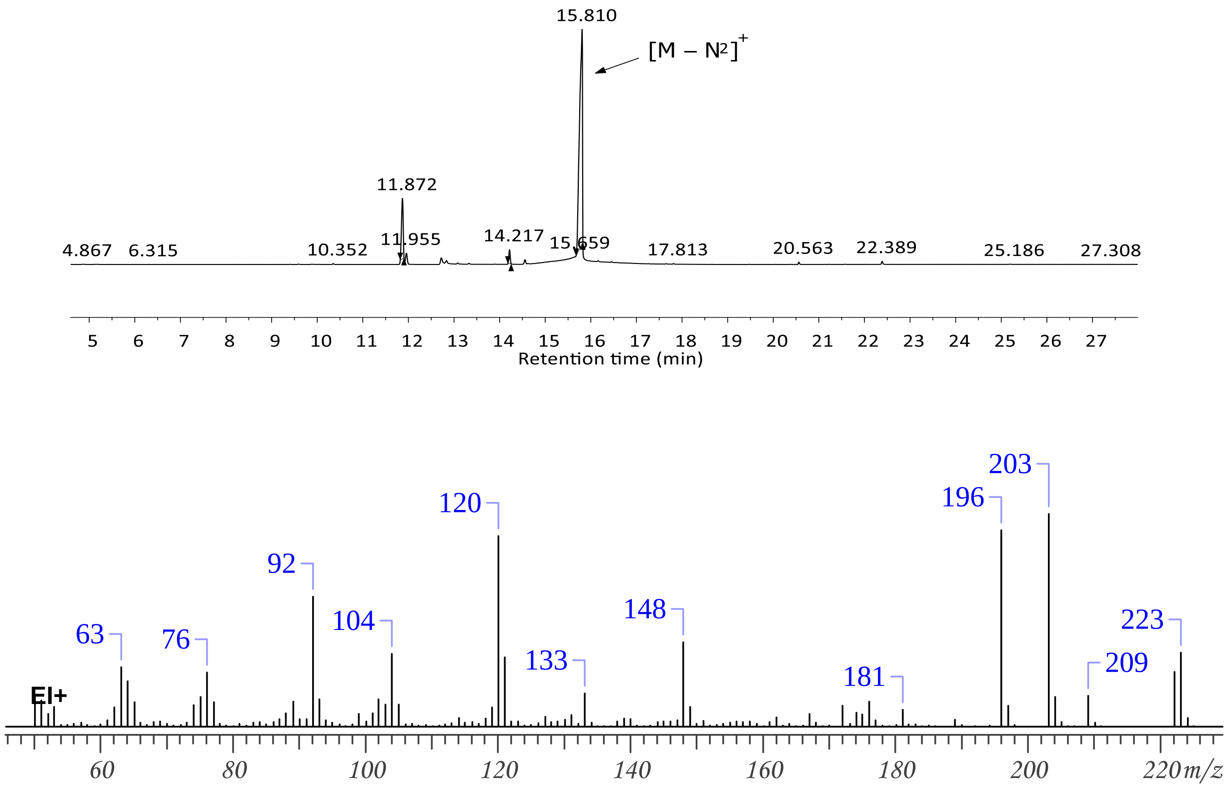

Figure S24. GC-MS of **4**.

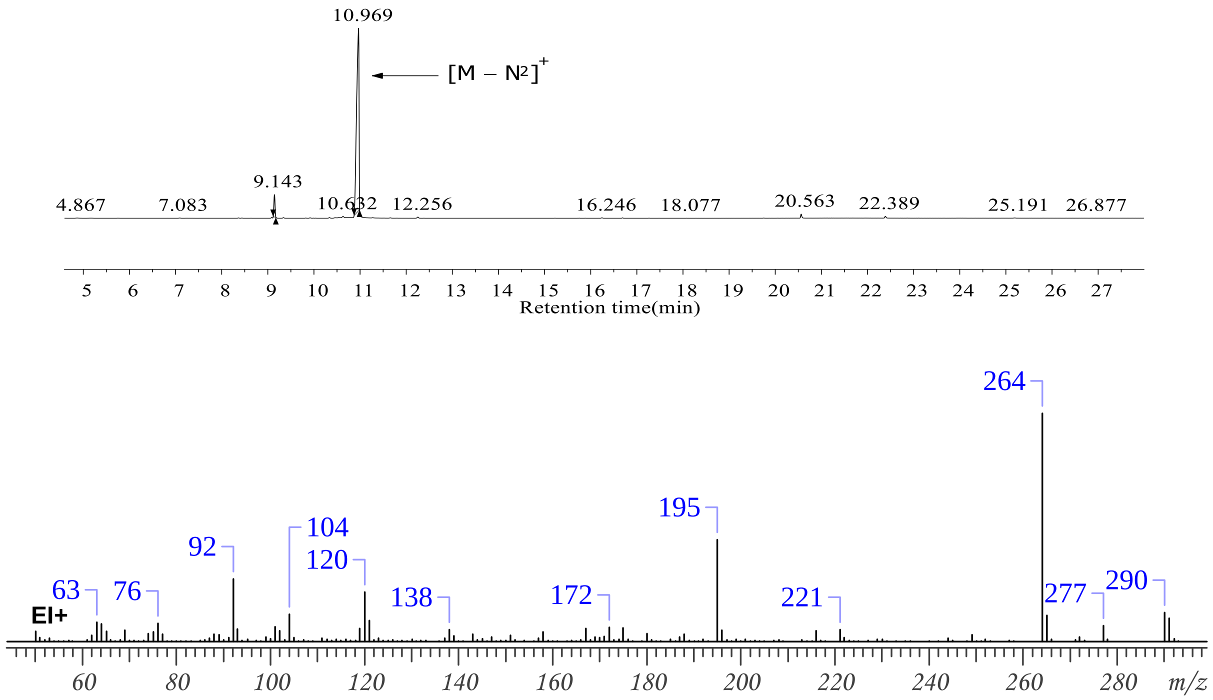

Figure S25. GC-MS of **5**.

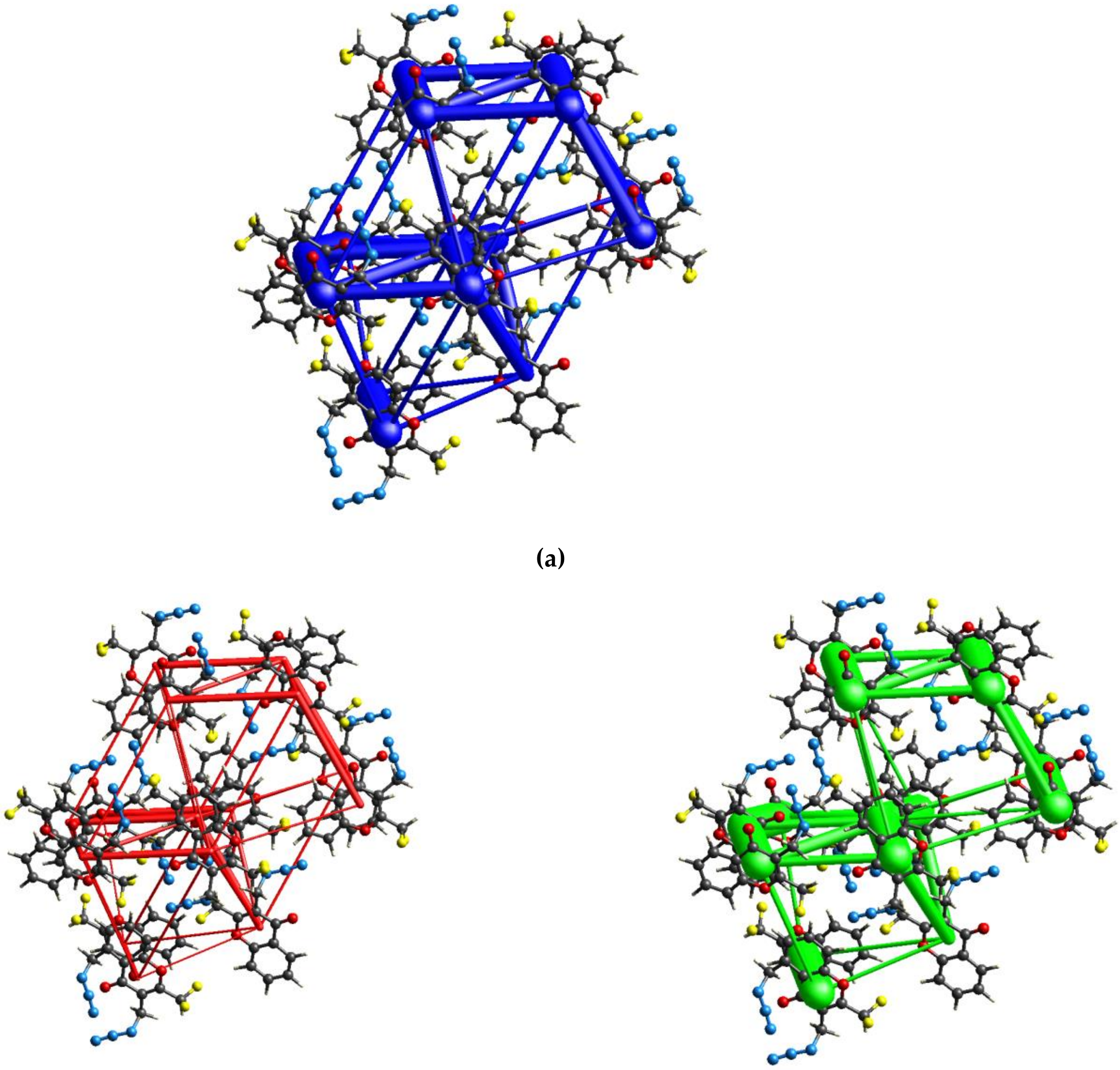

(a)

(b) **(c)**

Figure S26. Crystal lattice energy analysis applying the CE-B3LYP/6-31G(d,p) energy model and figures of energy frameworks for **4.** (**a**) Total Energy (**b**) Electrostatic Energy, (**c**) Dispersive Energy.
